# Genetic and environmental influences on structure of the social brain in childhood

**DOI:** 10.1016/j.dcn.2020.100782

**Published:** 2020-05-05

**Authors:** Mara van der Meulen, Lara M. Wierenga, Michelle Achterberg, Nadieh Drenth, Marinus H. van IJzendoorn, Eveline A. Crone

**Affiliations:** a Leiden Consortium on Individual Development, Leiden University, the Netherlands; b Institute of Psychology, Leiden University, the Netherlands; c Leiden Institute for Brain and Cognition, Leiden University, the Netherlands; d Department of Radiology, Leiden University Medical Center, the Netherlands; e Department of Psychology, Education and Child Studies, Erasmus University Rotterdam, the Netherlands; f School of Clinical Medicine, University of Cambridge, UK

**Keywords:** Social brain, Surface area, Cortical thickness, Heritability, Twins, Prosocial behavior, Empathy, Middle childhood

## Abstract

Prosocial behavior and empathy are important aspects of developing social relations in childhood. Prior studies showed protracted structural development of social brain regions associated with prosocial behavior. However, it remains unknown how structure of the social brain is influenced by genetic or environmental factors, and whether overlapping heritability factors explain covariance in structure of the social brain and behavior. The current study examined this hypothesis in a twin sample (aged 7–9-year; N = 512). Bilateral measures of surface area and cortical thickness of the medial prefrontal cortex (mPFC), temporo-parietal junction (TPJ), posterior superior temporal sulcus (pSTS), and precuneus were analyzed. Results showed genetic contributions to surface area and cortical thickness for all brain regions. We found additional shared environmental influences for TPJ, suggesting that this region might be relatively more sensitive to social experiences. Genetic factors also influenced parent-reported prosocial behavior (A = 45%) and empathy (A = 59%). We provided initial evidence that the precuneus shares genetically determined variance with empathy, suggesting a possible small genetic overlap (9%) in brain structure and empathy. These findings show that structure of the social brain and empathy are driven by a combination of genetic and environmental factors, with some factors overlapping for brain structure and behavior.

## 1 Introduction

Developing and maintaining social relations with others is often dependent on prosocial behavior, which can be defined as voluntary behaviors intended to benefit another individual (e.g. helping and sharing; Eisenberg et al., 2006). Many prior studies have investigated the origins of prosocial behavior in children and adolescents, using multiple indices such as self-report (van de Groep et al., 2018; Vrijhof et al., 2016), parent-report (Knafo-Noam et al., 2015; Thijssen et al., 2015), and experimental measures (Fehr et al., 2008). These studies showed that the first signs of prosocial behavior are already apparent in 18-month old children (Warneken and Tomasello, 2006), but at the same time this behavior continues to develop over childhood and adolescence (Eisenberg et al., 2006; Güroğlu et al., 2014). This leads to the question whether prosocial behavior is inherently present or whether this behavior is learned through social experiences (Blakemore and Mills, 2014).

One approach to investigate the factors that may contribute to prosocial behavior is by examining the neural processes that underlie social behaviors. Researchers have demonstrated a distinct set of brain regions (known as the “social brain”) that are recruited during (pro)social thoughts and actions using functional neuroimaging, including the medial prefrontal cortex (mPFC), temporal parietal junction (TPJ; Blakemore, 2008; Burnett and Blakemore, 2009; Gunther Moor et al., 2012; Will et al., 2015), posterior superior temporal sulcus (pSTS; Blakemore, 2008; Frith and Frith, 2003), and precuneus (Carrington and Bailey, 2009). Interestingly, at the structural level these brain regions continue to develop throughout childhood and adolescence (Mills et al., 2014), but it is currently unknown to what extent the development of these specific regions is biologically programmed or sensitive to environmental influences. Also, no study to date examined the genetic and environmental influences on the social brain in relation to prosocial behavior.

This question can be examined in more detail by using a twin design that allows for distinguishing between genetic and environmental influences. By comparing behaviors of monozygotic twins (who share 100 % of their genes) with dizygotic twins (who share on average 50 % of their genes), it is possible to unravel whether processes are more strongly driven by additive genetic factors, shared environment (family-related factors), or unique environment (child-specific factors; McLoughlin et al., 2007; Plomin et al., 2001). Prior studies using this approach showed that global and regional measures of brain structure are strongly sensitive to genetic effects (Jansen et al., 2015; Peper et al., 2007; Teeuw et al., 2018). However, to date heritability of distinct regions in the social brain has not yet been investigated. These regions are of specific interest, given that they support social behaviors, and therefore may be more open to environmental and social experiences (Blakemore and Mills, 2014). A prior study by Mills et al. (2014) distinguished between three indices of brain structure: cortical thickness, surface area and cortical volume (the latter being the product of thickness and surface area) and focused on the key regions in the social brain typically involved in social behavior (Blakemore, 2012). They showed that cortical volume development of the mPFC, TPJ, and pSTS follows a cubic trajectory, peaking around age 9. In contrast, development of cortical thickness showed linear decreases from childhood into adolescence, whereas development of surface area shows a cubic trajectory, similar to cortical volume, with different peaks for mPFC (around age 8), TPJ (around age 11), and pSTS (around age 13). These findings converge with prior studies showing that cortical thickness and surface area have distinct developmental patterns (Gilmore et al., 2018; Raznahan et al., 2011; Tamnes et al., 2017; Vijayakumar et al., 2016; Wierenga et al., 2014). In the current study we advance these findings by examining genetic and environmental influences on structural measures of four regions in the social brain network: the mPFC, TPJ, pSTS, and precuneus. We specifically focus on middle childhood as this is an important developmental stage for social development, with children engaging in social interactions outside the family context (Del Giudice et al., 2009). Additionally, middle childhood is a transition period to the pronounced grey matter changes of adolescence (Mills et al., 2016; Wierenga et al., 2014). We examined surface area and cortical thickness separately, as prior studies showed that surface area is more susceptible to varying environmental influences than cortical thickness (Noble et al., 2015), which in turn is consistent with the finding that surface area growth showed more individual differences than cortical thickness growth (Mills et al., 2014). It should be noted that some other studies demonstrated that shared environmental influences, such as SES, are larger for changes in cortical thickness rather than changes in surface area (Piccolo et al., 2016). It therefore remains an unanswered question whether both measures of social brain structure are, in addition to genetic influences, also sensitive to shared environmental influences.

Heritability studies on prosocial behavior revealed that prosocial behavior as indicated by parent-report is strongly influenced by genetics in children at the age of seven years, with heritability estimates ranging from 60 to 69 % (Knafo-Noam et al., 2015; Knafo and Plomin, 2006). An experimental study using a prosocial compensation task in 7–9-year-old children, however, did not find significant genetic nor shared environmental influences (van der Meulen et al., 2018). Nevertheless, studies with adolescents showed that prosocial behavior is sensitive to peer pressure, suggesting that the environment can also impact prosocial behavior (Foulkes et al., 2018). Possibly, these different findings are due to use of different methods to measure prosocial behavior, with parent-report measuring prosocial behavior across contexts, and experimental tasks measuring prosocial behavior in a specific situation. Furthermore, constructs closely related to prosocial behavior such as empathy (an emotional reaction that is elicited by another individual’s emotional response) and perspective taking (the ability to understand and perceive the motives, ideas, and wishes of others; Penner and Finkelstein, 1998) should be taken into account when estimating heritability. Specifically, Knafo et al., 2008 showed that empathy and prosocial behavior share genetic and unique environmental influences in childhood. Therefore, in this study we focused on both parent-reported prosocial behavior and empathy in relation to structural estimates of the social brain.

So far there is little understanding of the underlying biological processes driving prosocial behavior in middle childhood, and only two cross-sectional studies have focused on associations between brain structure and prosocial behavior in children. Wildeboer et al. (2018) found a positive association between cortical thickness of the pars orbitalis and pre- and post-central cortex and costly donating behavior in 8-year-old children. In addition, Thijssen et al. (2015) found positive associations between cortical thickness of the mPFC and precuneus and parent-reported prosocial behavior in a large sample of 6-9-year-old children. To elaborate on these initial brain-behavior associations in children, we used the novel approach of simultaneously investigating unique as well as shared genetic and environmental influences on structure of the social brain and prosocial behavior in middle childhood. Moreover, we used regions that are well established for their functional contribution to social behavior, and that are based on a template that is replicable across studies.

Taken together, in the current study we investigated heritability of prosocial behavior and structure of the social brain (mPFC, TPJ, pSTS and precuneus) in a large middle childhood twin sample (N = 512, aged 7–9). The aims for this study were twofold. Our first aim was to examine the extent to which variance in both prosocial behavior and structure of the social brain was accounted for by genetics, shared and unique environment (Knafo-Noam et al., 2015; Panizzon et al., 2009). Since this is one of the first studies to investigate heritability of the social brain in middle childhood, we also included two control regions (cuneus and lingual gyrus) in our heritability analyses to check the specificity our findings. Within the structural measures of the social brain, we examined estimates of heritability for cortical thickness and surface area separately (Winkler et al., 2010). Our second aim was to explore whether covariance in prosocial behavior and structure of the social brain was accounted for by overlapping genetic factors. In addition, we studied whether covariance in prosocial behavior and empathy was accounted for by overlapping genetic factors in middle childhood (Knafo et al., 2008).

## 2 Methods

### 2.1 Participants

Participants were recruited for the longitudinal twin study of the Leiden Consortium on Individual Development (L-CID; also see Euser et al., 2016). We obtained address information through municipal registries and invited families with twin children (born between 2006–2008) to participate. Same-sex twin pairs were included in the study when they were 7–9 years old at the time of data collection, had normal (or corrected to normal) vision, were fluent in Dutch or English, and did not suffer from psychological or physical conditions that could hinder their performance on the tasks. The study was approved by the Dutch Central Committee on Research Involving Human Subjects (CCMO) and parental informed consent was obtained before data collection. Parents received financial compensation (€80) for their time invested in the study and children received a small gift.

We initially included 512 participants (256 same-sex twin pairs) and their parents in the L-CID middle childhood cohort (previously described in Achterberg et al., 2018; van der Meulen et al., 2018). We distinguished between the primary parent (parent who reported spending most time with the children) and the other parent. Family characteristics of the sample are described in Table 1. This population sample included 11 participants diagnosed with an Axis-I disorder; nine participants were diagnosed with attention deficit hyperactivity disorder (ADHD) and/or attention deficit disorder (ADD), one participant was diagnosed with pervasive developmental disorder not otherwise specified (PDD-NOS), and one participant was diagnosed with generalized anxiety disorder (GAD). For information on psychiatric disorders, we asked parents whether the children received a medical diagnosis from a psychologist or medical expert. Estimated participant IQ was within normal range (*M* = 103.58, *SD* = 11.76, *range* = 72.5–137.5; estimated via the subscales Block Design and Similarities of the Wechsler Intelligence Scale for Children, 3rd version (WISC-III); Wechsler (1991)). Twin zygosity was assessed using DNA information from buccal cell samples, collected via mouth swabs. Missing DNA information for one family was imputed with zygosity estimates derived from the Zygosity Diagnosis Questionnaire (Rietveld et al., 2000).

**Table 1:** Family characteristics of the L-CID middle childhood cohort.

Marital status parents	
Married	69%
Registered partnership	5%
Living together	19%
Single	7%

	Primary parent	Other parent
*Parent-child relationship*		
Biological parent	99%	96%
Adoptive parent	1%	1%
Step-parent		3%

Education level parent		
Primary education	1%	1%
Secondary vocational education	5%	7%
Secondary higher/pre-university education; tertiary vocational education	29%	31%
College; university bachelor education	42%	36%
Post-college education; university master education	23%	25%

**Fig. 1:**
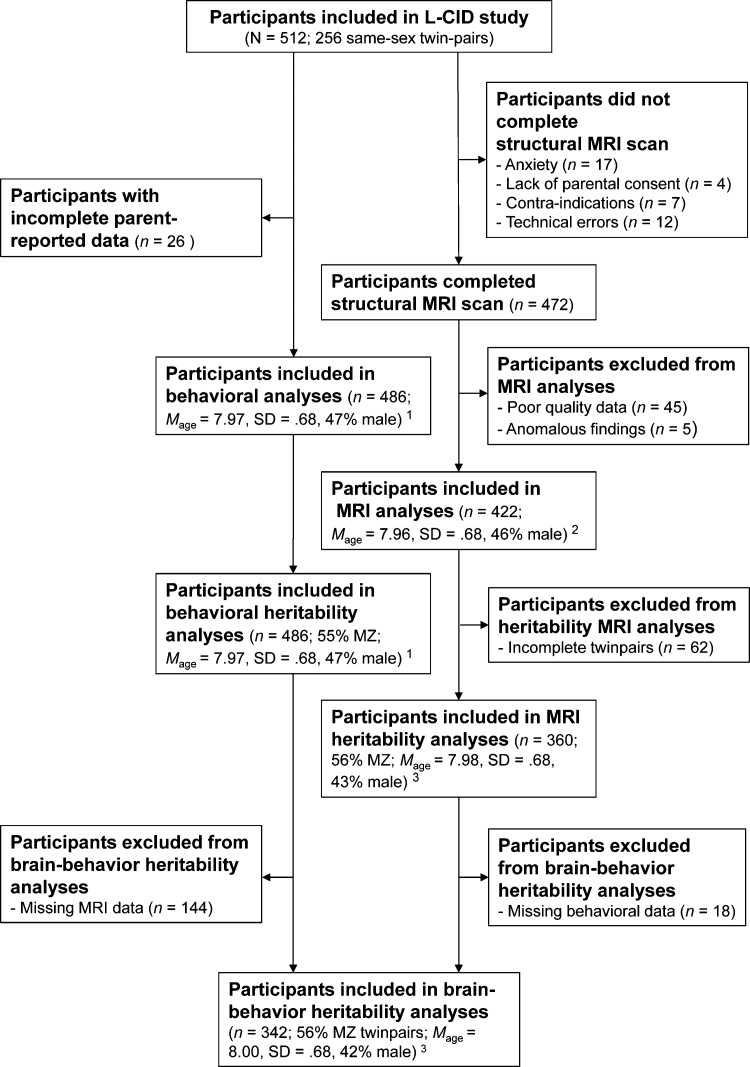
Flowchart of inclusion of samples (including demographic information) at various stages of the study. *Note*. MZ = monozygotic twin pairs; ^1^ Diagnosed Axis-I disorders: ADHD and/or ADD (eight participants), PDD-NOS (one participant), generalized anxiety disorder (GAD; one participant); ^2^ Diagnosed Axis-I disorders: ADHD and/or ADD (six participants), PDD-NOS (one participant), GAD (one participant); ^3^ Diagnosed Axis-I disorders: ADHD and/or ADD (five participants), PDD-NOS (one participant), GAD (one participant).

Of the initial 512 participants, 40 participants did not complete the structural MRI scan. An additional 45 participants had poor quality data and five participants had anomalous findings. Therefore we included 422 participants (including 180 complete twinpairs) in our MRI analyses. Out of 512 participants, 26 participants did not have complete parent-reported data, so we included 486 participants (including 243 complete twinpairs) in our behavioral analyses. We included 342 participants (171 complete twinpairs) with complete MRI and behavioral data in our bivariate heritability analyses. An overview of the participants included in analyses at various stages of the study can be found in Fig. 1.

### 2.2 Procedure

Both parents were asked to fill out several questionnaires before the lab visit. During the lab visit (described in detail in Achterberg and van der Meulen (2019) and Crone et al. (this issue)), participants were thoroughly prepared for the MRI procedure, by receiving extensive explanations and a practice session in a mock scanner. During the scanning session a high resolution structural scan was collected. All participants were scanned on the same scanner.

### 2.3 MRI data acquisition and processing

All MRI scans were acquired on a Philips Ingenia MR 3.0 T scanner at the Leiden University Medical Center, using a standard 32-channel whole-head coil. A high resolution 3D T1-weighted anatomical image was collected (TR =9.8 ms, TE =4.6 ms, 140 slices, voxel size = 1.17 × 1.17 × 1.2 mm, and FOV = 224 × 177 × 168 mm). In order to reduce motion artifacts, foam inserts were used within the head coil to restrict head movement. In addition, participants were instructed to watch a child-appropriate movie during the T1-weighted scan acquisition in order to decrease head motion (Greene et al., 2018). Furthermore, to increase scan quality T1-weighted scans were visually inspected on motion artifacts during the scanning session (i.e. visible movement rings) and repeated if motion was detected (6% of participants).

Next, T1-weighted images without anomalous findings were processed in FreeSurfer (v5.3.0). Tissue classification and anatomical labeling was performed using the well-validated and well-documented FreeSurfer v5.3.0 software (http://surfer.nmr.mgh.harvard.edu/). In short, this software includes non-brain tissue removal (Clarkson et al., 2011; Ségonne et al., 2004), segmentation of deep gray matter (Fischl et al., 2004a, 2004b; Hutton et al., 2009; Salat et al., 2004), intensity normalization (Sled et al., 1998), and correction of gray-white matter boundary topology (Fischl et al., 2001; Segonne et al., 2007).

**Fig. 2:**
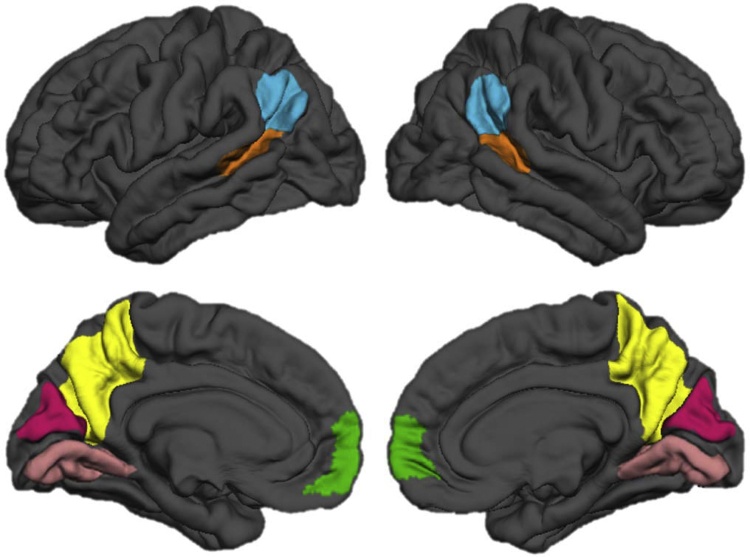
Regions of interest in the social brain, including TPJ (blue), pSTS (orange), mPFC (green), and precuneus (yellow). Cuneus (magenta) and lingual gyrus (pink) were included as control regions. Left side of the panel indicates left hemisphere, right side of the panel indicates right hemisphere (For interpretation of the references to colour in this figure legend, the reader is referred to the web version of this article).

For three of the regions of interest (mPFC, TPJ, and pSTS; see Fig. 2), we used the templates described in Mills et al. (2014) (available on https://figshare.com/articles/Social_Brain_Freesurfer_ROIs/726133) for each T1-weighted scan, to increase replicability across studies. Note that we did not include the anterior temporal cortex (included as another region of interest in the study by Mills et al. (2014)) as cortical reconstruction of this region was unsuccessful for one or both hemispheres in a large number of participants (45% of sample). Additionally, the precuneus was derived from the Desikan-Killiany atlas (Desikan et al., 2006). For our specificity analyses we included cuneus and lingual gyrus from the Desikan-Killiany atlas. For each labeled structure, we extracted measurements of surface area (in mm^2^) and cortical thickness (in mm) for left and right hemisphere separately. To reduce the number of statistical tests, we combined structural measures for each hemisphere. All lateralized results are presented in the supplementary files. To compute bilateral measurements of surface area we averaged measurements for left hemisphere (lh) and right hemisphere (rh) surface area (SA): (lh SA+rh SA)/2. To compute bilateral measurements of average cortical thickness (CT), we took the size of each region into account (also see Bos et al., 2018) by using the following formula:

$$\frac{\left(lh\;CT*lh\;SA)+(rh\;CT*rh\;SA\right)}{\left(lh\;SA+rh\;SA\right)}$$

### 2.4 Quality control of T1-weighted scans

To establish the quality of the T1-weighted scans, we manually rated whether cortical reconstruction was of sufficient quality based on a set of specific criteria (e.g. movement, missing brain areas in reconstruction, inclusion of dura or skull in reconstruction) for each FreeSurfer processed scan. Three raters were trained to perform manual quality control using 20 scans from an independent dataset. Details regarding this procedure are described in the supplementary information of Klapwijk et al. (2019). In short, for every scan we inspected i) whether pial surface and gray/white matter boundaries had been correctly defined by Freesurfer, ii) whether the scan was affected by movement, iii) whether any brain areas were missed during reconstruction and iv) whether dura or skull was included in the reconstruction. Based on these assessment criteria, we rated the quality of each scan. For a large number of participants (45%), cortical reconstruction had failed for the anterior temporal lobes. That is to say, sections of grey matter of the anterior temporal lobes had not been included in the pial surface reconstruction by FreeSurfer. Therefore we did not include the anterior temporal lobes in our assessments of scan quality (e.g. scans with failed reconstruction of the anterior lobes that had correct reconstruction of all other brain regions were included in our analyses). Out of 467 total scans, 422 scans (91 %) were rated to be of sufficient quality and 45 scans (9 %) were rated to be of insufficient quality. Scans rated to be of insufficient quality were excluded from further analyses.

### 2.5 Parent-reported prosocial behavior

To measure parent-reported prosocial behavior we used subscales of two different questionnaires: the 5-item “Prosocial” subscale of the Strengths and Difficulties Questionnaire (SDQ; Goodman, 1997), and the 13-item “Empathic and Prosocial Response to Another’s Distress” subscale of the My Child Questionnaire (MC; Kochanska et al., 1994). The SDQ subscale was answered with a 3-point Likert scale (1 = not true, to 3 = certainly true), and included items such as *“My child is considerate of other people’s feelings”*. The MC subscale was answered with a 5-point Likert scale (1 = not true, to 5 = true) and included items such as *“My child will try to comfort or reassure another in distress”*. For most participants (74 %) both parents completed the questionnaires on prosocial behavior, whereas for a smaller group of participants (21%) one of the parents completed the questionnaires. Parent-report was missing for the remaining participants (5 %).

The 18 items were factor analyzed using principal component analysis with Varimax rotation. To prevent within-twin dependence in the PCA, we randomly divided co-twins over two samples (A and B), such that one co-twin of each twin-pair was allocated to sample A and the other twin was allocated to sample B. Scores on the SDQ were recoded (from 1-2-3 to 1-3-5) in order to create a scale comparable to the scores on the MC (range 1–5). First, we ran the PCA on the items answered by one of the parents in sample A. KMO (.81) and Bartlett’s test (*X^2^* (153) = 1185.21, *p* < .001) indicated that the 18 items were suitable for PCA. Our analysis yielded two factors. The first factor (explaining 26.15 % of the variance) was labeled ‘Prosocial’ and had high loadings for items such as *“My child shares readily with other children”*. The second factor (explaining 13.11 % of the variance) was labeled ‘Empathy’ and had high loadings for items such as *“My child is upset by stories in which characters are hurt or die”*. Two items did not fit well with either of the two components: *“My child may occasionally tease a pet if unsupervised”* (recoded) and *“My child feels good when good things happen to movie characters”*. These items were not included in further analyses (see Supplementary Table S1 for an overview of the final subscale composition). We found a similar component structures with the other parent in sample A, and for both parents in sample B, indicating that this outcome was fitting for all participants and parents in our sample. Subscale scores were calculated by computing the mean of the items. We found positive correlations between both parents on the subscale ‘Prosocial Behavior’ (sample A: *r* = .48; sample B: *r* = .53, *p*’s<.001) and ‘Empathy’ (sample A: *r* = .37; sample B: *r* = .43, *p*’s<.001). Therefore, we created two new variables by calculating the mean rating of both parents for the subscale ‘Prosocial Behavior’ and for the subscale ‘Empathy’. For both subscales, a higher score indicated more prosocial behavior or empathy. For completeness, we reported correlation coefficients between the mean factor scores of the newly created subscales and the original subscales of SDQ and MC (separately for sample A and B) in Supplementary Table S2.

### 2.6 Data analysis

Analyses were performed in SPSS (version 23.0; IBM SPSS Statistics, IBM Corporation) and R (version 3.3.2; R Core Team, 2015). Outliers (z-value < -3.29 or > 3.29) detected in parent-reported prosocial behavior, surface area of mPFC and lingual gyrus, and cortical thickness of TPJ were winsorized (Tabachnick and Fidell, 2013). In order to take into account effects of age, sex, and IQ on behavior and structural measures of the social brain, we performed regression analyses on all outcome measures, with age, sex, and IQ as predictor variables, separately for sample A and B (for regression results see Supplementary Table S3). We then used the unstandardized residuals as variables in our subsequent analyses.

To test heritability estimates for structural properties of the social brain, prosocial behavior, and empathy we first computed within-twin pair Pearson correlations for each outcome variable, separately for MZ (monozygotic) and DZ (dizygotic) twins. Since MZ twins share 100% of their genes, and DZ twins only share around 50% of their genes, a high MZ correlation would indicate influence of genetic factors. A DZ correlation higher than half the MZ correlation would indicate influence of shared environment (Knafo-Noam et al., 2015). MZ and DZ within-twin correlations coefficients smaller than 1 indicate additional effects of (unique) environment. We tested whether within-twin correlations were significantly different for MZ compared to DZ twins using Fisher r-to-z transformations. We used univariate ACE models to describe the relative contribution of genetic (A), shared environmental (C), and unique environmental factors and/or measurement error (E) to variance in brain structure and prosocial behavior, using the OpenMx package (version 2.7.4; Neale et al. (2016)) in R.

To explore which factors explain variance best, we next performed a set of post hoc tests. For each outcome variable, three additional models (AE, CE, and E) were estimated. The fit of each model was then compared to the fit of a more parsimonious model (e.g. ACE to AE) by subtracting the -2 log likelihood (-2LL), resulting in an estimate of the Log-Likelihood Ratio Test (LRT). The LRT follows the *X^2^* distribution. The model with the least number of parameters that did not fit significantly worse than the more complex model (as indicated by LRT < 3.84) was selected as the best fit. For models with equal numbers of parameters (i.e. AE and CE) the model with the lowest Akaike Information Criterion (AIC; Akaike (1974)) was selected. As ACE models were used to describe heritability estimates and explorative post-hoc tests were used to determine model fitting, we did not correct for multiple comparisons.

To investigate shared heritability estimates we first inspected phenotypic brain-behavior associations using least square regressions with brain structure predicting prosocial behavior. In order to overcome the nested nature of twin data, we used heteroscedasticity-consistent standard error (HSCE) estimations from the HSCE macro (Hayes and Cai, 2007), using the HC3 method (Ervin and Long, 2000). Using the same heteroscedasticity-correcting method, we also tested the phenotypic association between prosocial behavior and empathy to further investigate shared heritability estimates for prosocial behavior and empathy. Results were Bonferroni-corrected for multiple testing, using a lowered threshold of α = .0021 for the 24 associations (α = 0.05/24).

**Fig. 3:**
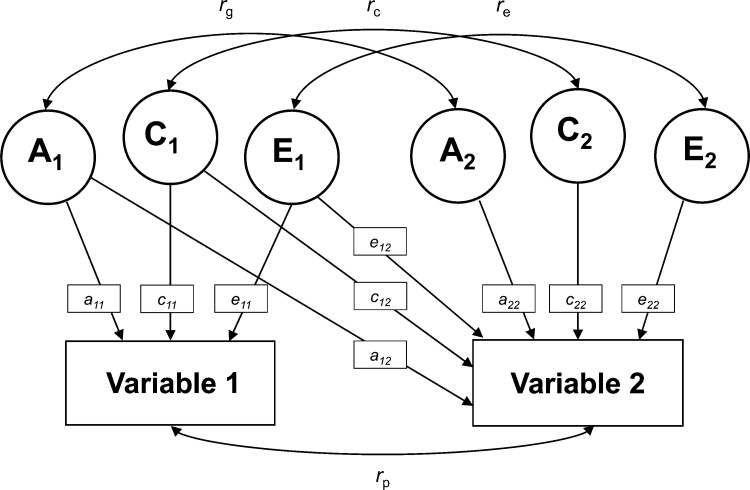
Bivariate ACE model, visualizing contributions of genetic (A), shared environmental (C) and unique environmental (E) on two variables. Paths a_11_-e_11_ and a_22_-e_22_ indicate contributions of genes and environment on variables 1 and 2, respectively. Paths a_12_-e_12_ indicate contributions of the factors for variable 1 to variable 2. *r*_g_ = genetic correlation, *r*_c_ = shared environmental correlation, *r*_e_ = unique environmental correlation, *r*_p_ = covariance.

Finally, we used bivariate ACE models to describe the relative contribution of genetic (A), shared environmental (C), and unique environmental factors/measurement error (E) to covariance between structural measures of the social brain, prosocial behavior, and empathy, using the OpenMx package (version 2.7.4; Neale et al. (2016)) in R. We performed a bivariate Cholesky decomposition model (see Fig. 3), a base model for bivariate analyses (Neale and Cardon, 1992; Verweij et al., 2012). First a saturated Cholesky model was estimated, and next an ACE model was estimated. Similar to the univariate heritability analyses, we then performed a set of post hoc tests to test whether an ACE, AE, CE or E model would best explain covariance. The fit of each model was compared to the fit of a less complex model (e.g. ACE to AE) using the LRT and AIC. After selecting the best fitting model, standardized path loadings were computed and squared to estimate the relative contribution of A, C, and E on variance in brain structure and behavior. Next, we used the best-fitting model to estimate contributions of genes, shared and unique environment/measurement error to covariance (*r*_p_) between brain structure and prosocial behavior (Plomin et al., 2001). The contribution of genes to the covariance was computed with the following formula:

$$\frac{estimate\;path\;a11*estimate\;path\;a12}{covariance}$$

using the standardized path loadings (Treur et al., 2016). Contributions of shared and unique environment/measurement error to covariance were computed using the path loadings for paths *c* and *e*, respectively. Finally, genetic (*r*_g_) and environmental correlations (*r*_c_ and *r*_e_) were obtained from the correlation matrix of the best fitting model to quantify the extent to which brain structure and prosocial behavior are influenced by overlapping genetic and environmental factors. It should be noted that the heritability of both brain structure and prosocial behavior could be high, but the genetic correlation between them could be low, indicating that different genetic factors influence brain structure and prosocial behavior.

## 3 Results

### 3.1 Univariate heritability of brain structure, prosocial behavior, and empathy

First we addressed to what extent genetic, shared environment and unique environmental factors contribute to variation in structural measures of the social brain, prosocial behavior, and empathy.

For brain structure (*n* = 180 twinpairs, 56% MZ) we examined genetic, shared environment and unique environment contributions for surface area and cortical thickness of mPFC, pSTS, TPJ, and precuneus separately. Within-twin correlations are presented in Table 2 and Supplementary Figure S1. For surface area, we found contributions of genetic factors for mPFC (A = 48 %), pSTS (A = 61 %) and precuneus (A = 81 %), as well as a small contribution of shared environmental factors for pSTS (C = 4 %). The remaining variance was best explained by unique environment/measurement error. Submodel fitting indicated that an AE model was best fitting for surface area of mPFC, pSTS and precuneus (see Supplementary Table S4). For surface area of TPJ, both genetic factors (A = 25 %) and shared environmental factors (C = 21 %) contributed, and submodel fitting indicated that no clear distinction could be made between an AE and CE model. However, the confidence interval of the E factor (39–68 %) in the full ACE model did not reach 100 %, so it is likely that familial influences (defined as the combination of genetic and/or shared environmental factors) are present. Our specificity analyses indicated that surface area of cuneus and lingual gyrus was best explained by genetic factors (A = 70 % for both regions), with a minor influence of shared environmental influence (C = 2 % and C = 1 %, respectively), with the remaining variance best explained by unique environmental factors/measurement error. Submodel fitting indicating that an AE model was best fitting for surface area of cuneus and lingual gyrus.

**Table 2:** Within-twin correlations and estimated contributions of genes (A), shared environment (C), and unique environment/measurement error (E). 95% confidence intervals for each estimate are provided between parentheses.

Outcome variable	rMZ	rDZ	Z	A²	C²	E²
*Surface area*						
mPFC	.48***	.21	2.03*	0.48 (0.32-0.61)	0.00 (†-0.28)	0.52 (0.39-0.68)
TPJ	.41***	.37**	.31	0.25 (†-0.60)	0.21 (0.00-0.49)	0.53 (0.39-0.68)
pSTS	.62***	.38**	2.13*	0.61 (0.23-0.72)	0.04 (†-0.36)	0.35 (0.26-0.47)
Precuneus	.82***	.28*	5.7***	0.81 (0.73-0.86)	0 (†-0.19)	0.19 (0.14-0.27)
Cuneus	.74***	.36**	3.76***	0.70 (0.32-0.79)	0.02 (†-0.36)	0.29 (0.21-0.39)
Lingual	.73***	.33**	3.84***	0.70 (0.59-0.79)	0.01 (†-0.34)	0.29 (0.21-0.39)

*Cortical thickness*						
mPFC	.23*	.15	.54	0.17 (0.00-0.39)	0.05 (0.00-0.31)	0.78 (0.61-0.96)
TPJ	.31**	.31**	.00	0.09 (0.00-0.48)	0.23 (0.00-0.42)	0.68 (0.52-0.84)
pSTS	.36***	.14	1.55	0.23 (0.00-0.43)	0.05 (0.00-0.37)	0.72 (0.57-0.88)
Precuneus	.55***	.21	2.66**	0.55 (0.26-0.67)	0.00 (0.42-0.22)	0.45 (0.33-0.61)
Cuneus	.71***	.36**	3.35***	0.73 (0.44-0.80)	0.00 (0.00-0.26)	0.27 (0.20-0.37)
Lingual	.60***	.08	4.02***	0.56 (0.35-0.68)	0.00 (0.00-0.16)	0.44 (0.32-0.59)

*Parent report*						
Prosocial behavior	.37***	.08	2.36*	0.45 (0.24-0.57)	0.00 (†-0.14)	0.55 (0.43-0.70)
Empathy	.75***	.42***	4.02***	0.59 (0.39-0.79)	0.15 (0.00-0.41)	0.27 (0.20-0.35)

rMZ = within-twin correlation for monozygotic twins, rDZ = within-twin correlation for dizygotic twins.

* *p* < .05; ** *p* < .01; *** *p* < .001. Significant Z-scores indicate significant differences between MZ and DZ associations.† The 95% confidence interval bounds could not be estimated reliably.

With respect to cortical thickness, estimations for contributions of genetics, shared environment and unique environment showed a substantial contribution of genetics for precuneus (A = 55 %), and the remaining variance was best explained by unique environment/measurement error. Submodel fitting indicated that an AE model was best fitting for cortical thickness of precuneus (see Supplementary Table S4). For cortical thickness of mPFC, TPJ, and pSTS both genetic (A = 17 %, 9 %, and 23 %, respectively) and shared environmental factors (C = 5 %, 23 %, and 5 %, respectively) contributed, and submodel fitting indicated that no clear distinction could be made between an AE and CE model. The confidence interval of the E factor did not include 100% however, providing room for familial influences. Our specificity analyses indicated that cortical thickness of cuneus and lingual gyrus was best explained by genetic factors (A = 73 % and A = 56 %, respectively) with the remaining variance best explained by unique environmental factors/measurement error. Submodel fitting indicating that an AE model was best fitting for cortical thickness of cuneus and lingual gyrus. For completeness, lateralized within-twin correlations and heritability estimates for surface area and cortical thickness are presented in Supplementary Tables S5 (left hemisphere) and S6 (right hemisphere).

Finally, in the behavioral heritability analyses (*n* = 243 twinpairs, 55 % MZ) we found substantial contributions of genetics for prosocial behavior (A = 45 %) and empathy (A = 59 %), in addition to a smaller contribution of shared environment to empathy (C = 15 %). The remaining variance was best explained by unique environment/measurement error. Submodel fitting indicated that an AE model was best fitting for both prosocial behavior and empathy (see Supplementary Table S4).

### 3.2 Bivariate heritability of brain structure, prosocial behavior, and empathy

First we investigated phenotypic brain-behavior associations as a starting point for our bivariate heritability analyses. We found a significant negative association between cortical thickness of precuneus and empathy (*β* = −.82, *t*(396) = -3.15, *p* = .002). All other brain-behavior associations were not significant (also see Table 3). Lateralized brain-behavior associations are reported in Supplementary table S7. Additionally, we found a significant positive association between prosocial behavior and empathy (*β* = .45, *t*(482) = 6.96, *p* < .001). Both significant associations survived Bonferroni-correction (α_corrected_ = .0021).

**Table 3:** Phenotypic brain-behavior associations between structural brain measures, prosocial behavior, and empathy.

	Prosocial behavior		Empathy	
	*β*	*p*	*β*	*p*
*Surface Area*				
mPFC	.00	.35	.00	.41
TPJ	.00	.77	.00	.75
pSTS	.00	.43	.00	.99
Precuneus	.00	.61	.00	.42
Cuneus	.00	.68	.00	.83
Lingual	.00	.54	.00	.55

*Cortical Thickness*				
mPFC	.00	.99	.00	.99
TPJ	−.27	.04	−.29	.12
pSTS	−.05	.74	−.04	.85
Precuneus	−.28	.20	**−.82**	**.002**
Cuneus	−.20	.29	−.42	.08
Lingual	.03	.87	−.32	.15

*Behavior*				
Empathy	**.45**	**< .001**		

Significant associations are indicated in bold font.

We then tested the contributions of genetics, shared environment and unique environment to covariance between structure of the social brain and prosocial behavior using bivariate ACE models. Overall, path loadings in the bivariate ACE models were comparable to those of the univariate ACE models.

**Fig. 4:**
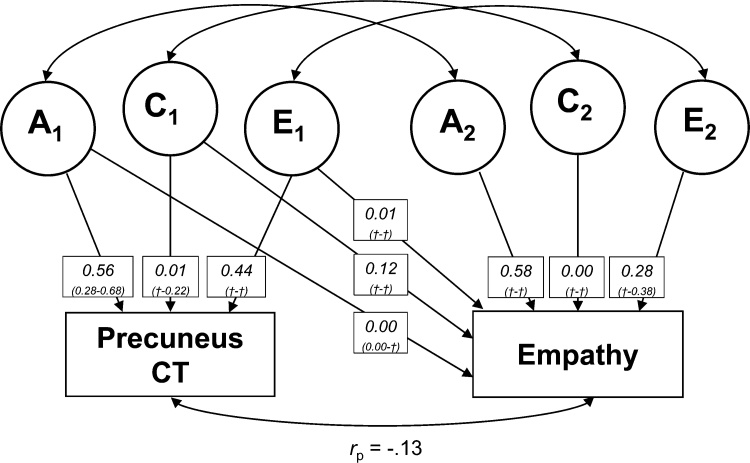
Visualization of bivariate ACE model with cortical thickness of precuneus and empathy. Numbers represent squared standardized path loadings, with 95% confidence intervals in parentheses († indicates unreliable estimation of the 95% confidence interval bounds).

We used a bivariate ACE model to describe shared variance between cortical thickness of precuneus and empathy (*n* = 171 twinpairs, 56% MZ). A substantial part of the variance in cortical thickness of precuneus (path *a*_11_ = .56) and empathy (path *a*_12_ + path *a*_22_ = .58) was best explained by genetics. The remaining variance was best explained by unique environment/measurement error. Standardized squared path loadings can be seen in Fig. 4. We found that a bivariate AE model was best fitting (see Supplementary Table S8 for full model comparisons). Based on the bivariate AE model, genetic effects explained 42% of the covariance between cortical thickness of precuneus and empathy (*r*_p_ = −.13), whereas 58% of the covariance was explained by unique environment/measurement error (also see Supplementary Table S9). Furthermore, we found that cortical thickness of precuneus and empathy were influenced by overlapping genetic (*r*_g_ = −.08) and unique environmental (*r*_e_ = -.20) factors. These results indicate that some genetic and unique environmental factors account for both lower cortical thickness of precuneus and higher empathy (or vice versa).

**Fig. 5:**
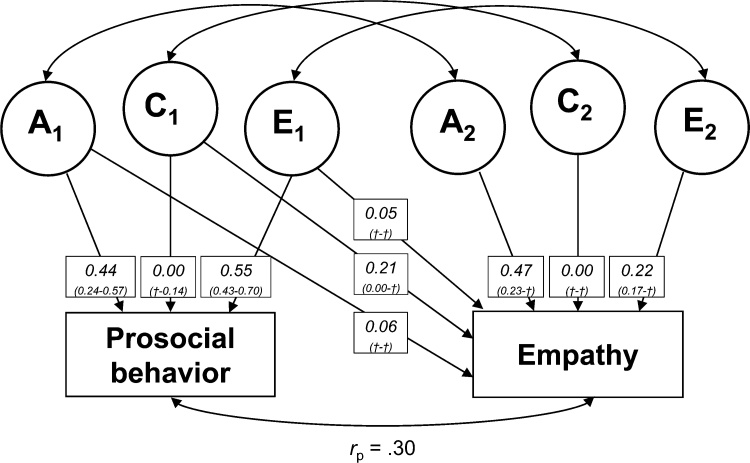
Visualization of bivariate ACE model with prosocial behavior and empathy. Numbers represent squared standardized path loadings, with 95% confidence intervals in parentheses. († indicates unreliable estimation of the 95% confidence interval bounds).

Finally, we used a bivariate ACE model to describe shared variance between prosocial behavior and empathy (*n* = 243 twinpairs, 55% MZ). A substantial part of the variance in prosocial behavior (path *a*_11_ = .44) and in empathy (path *a*_12_ + path *a*_22_ = .53) was best explained by genetics, with a smaller influence of shared environmental factors on empathy (path *c*_12_ + path *c*_22_ = .21). The remaining variance was best explained by unique environment/measurement error. Standardized squared path loading can be seen in Fig. 5. We found that a bivariate AE model was best fitting (see Supplementary Table S8 for full model comparisons). Based on the bivariate AE model, genetic effects explained 46% of the covariance between prosocial behavior and empathy (*r*_p_ = .30), whereas 54% of the covariance was explained by unique environment/measurement error (also see Supplementary Table S9). Furthermore, we found that prosocial behavior and empathy were influenced by overlapping genetic (*r*_g_ = .25) and unique environmental (*r*_e_ = .42) factors. These results indicate that overlapping genetic and unique environmental factors account for some of the variance in prosocial behavior and empathy.

## 4 Discussion

This study was driven by insights from prior studies showing protracted development of brain regions that are associated with prosocial behavior (mPFC, pSTS, TPJ and precuneus), but there is little understanding of what factors drive individual differences in the structure of these brain regions. The first aim of the current study was therefore to investigate the contribution of genetics and shared environment on the social brain, prosocial behavior, and empathy in 7–9-year-old children. We validated our findings of heritability of the social brain by including two control brain regions in our analyses. Second, we tested whether there was shared genetic and environmentally driven covariance in the social brain and prosocial behavior. In our analyses of brain structure, we distinguished between surface area and cortical thickness, as these may be differentially sensitive to environmental influences (Noble et al., 2015; Piccolo et al., 2016). For surface area, we found influence of genetic factors for mPFC, pSTS and precuneus, whereas environmental influences were more pronounced for TPJ. Additionally, we found a strong influence of genetics on cortical thickness of the precuneus, as well as influence of both genetics and environment on mPFC, TPJ and pSTS. In comparison, we found a pronounced influence of genetic factors on cuneus and lingual gyrus. On a behavioral level, we found that both prosocial behavior and empathy were strongly influenced by genetic factors. Finally, we found that covariance between cortical thickness of precuneus and empathy was partly explained by overlapping genetic factors. The discussion will first review the findings in social brain structure, followed by an interpretation of brain-behavior relations.

### 4.1 Genetic influences on structural properties of the social brain

Prior studies reported genetic influence on whole brain development in adults (Peper et al., 2007) and children (Peper et al., 2009; Teeuw et al., 2018), but this question was not yet addressed for regions in the social brain specifically, which have a prolonged developmental trajectory continuing until early adulthood for both cortical thickness and surface area (Mills et al., 2014). This led to the question whether the social brain was possibly more sensitive to influences from the environment (Blakemore and Mills, 2014).

Consistent with previous whole brain studies, there was evidence for genetic influences on brain structures in childhood, specifically for surface area, for all included regions in the social brain. Our estimates of genetic influence in mPFC, pSTS, and precuneus were comparable to prior studies that showed high estimates of genetic influence on global surface area (71–92%; Ma et al. (2016); Panizzon et al. (2009); Winkler et al. (2010)) and local surface area (including medial frontal regions; estimates ranging from 12 to 68 %) in adolescents and adults (Ma et al., 2016; Panizzon et al., 2009; Winkler et al., 2010). We found that variance in surface area of mPFC, pSTS and precuneus, as well as variance in the non-social brain regions (cuneus and lingual gyrus), was best explained by a combination of genetic factors and unique environment/measurement error.

In contrast to surface area, there was evidence for both genetic and environmental influences for cortical thickness of regions of the social brain in childhood, for all regions except for the precuneus. For the latter region, as well as for the control regions, a combination of genes and unique environment/measurement error best explained variance in cortical thickness. These findings complement previous studies that reported strong genetic influence on global cortical thickness (52–81%), but more variable estimates for local cortical thickness (0–76 %) across the lifespan (Lenroot et al., 2009; Panizzon et al., 2009; van Soelen et al., 2012; Winkler et al., 2010). Our heritability estimates for cortical thickness of the mPFC and pSTS indicated influences of both genetic and shared environmental factors, highlighting the possibility that cortical thickness of these social brain regions might be more susceptible to environmental influence compared to other brain regions (also see Blakemore and Mills (2014)).

Interestingly, the TPJ in particular showed a relatively high estimated influence of shared environment on both surface area and cortical thickness. The TPJ is consistently activated during social processing and social decision-making (Burnett et al., 2008; van der Meulen et al., 2016; van Hoorn et al., 2016a; van Hoorn et al., 2018). Given that social processing is dependent on environmental input, the TPJ might therefore be particularly sensitive to the social environment. In addition, the involvement of the TPJ in social behavior changes over development (Güroğlu et al., 2009, 2014; Güroğlu et al., 2011; Tousignant et al., 2017; Will et al., 2015) and this region often shows brain-behavior correlations in functional neuroimaging research (Van Hoorn et al., 2016b). Although structure of the TPJ follows similar developmental trajectories as other regions in the social brain (Mills et al., 2014) it is possible that differential genetic and environmental influences on structure of the TPJ become more pronounced over time, with environmental factors eventually having more impact on structure of the TPJ than genetic factors. Since we could not conclusively differentiate between sources of genetic or shared environmental influence on structure of the TPJ, longitudinal twin-studies are necessary to investigate this hypothesis, as previous research has indicated a change in heritability with age (Lenroot et al., 2009).

An important question we could not address in the current study is whether surface area or cortical thickness is more strongly influenced by environmental factors. According to the radial unit hypothesis (Rakic, 1995) surface area and cortical thickness are driven by different developmental processes, possibly providing room for different contributions of genetic and environmental processes. In the current data set, there was no clear pattern showing that either cortical thickness or surface area were more strongly influenced by the environment, although there was slightly more evidence for shared environment influences on cortical thickness. However, our current sample was too small to draw concrete conclusions.

### 4.2 Genetic influences on brain-behavior associations

An important aim of this study was to relate the structural brain measures to prosocial behavior, as this behavior is often associated with the functioning of the social brain (Blakemore, 2008). For this purpose we focused on parent-report measures of prosocial behavior and empathy, as these measures encompass multiple contexts (Carlo and Randall, 2002) and reporting complex social behaviors such as prosocial behavior and empathy might be challenging for children (Richaud et al., 2017). We found that both prosocial behavior and empathy show strong influences of genetics, which is consistent with earlier studies reporting high estimates of heritability for parent-reported prosocial behavior (39–69 %) and empathy (34–76 %; Gregory et al. (2009); Knafo-Noam et al. (2015); Knafo and Plomin (2006); Knafo et al. (2008); Melchers et al., 2016. Moreover, parent-reported prosocial behavior and empathy were positively associated, supporting previous findings of a multi-faceted perspective on prosocial behavior (Eisenberg et al., 2014; Knafo-Noam et al., 2015). We found that not all of the covariance between prosocial behavior and empathy could be attributed to overlapping genetic and unique environmental factors, in line with findings by Knafo et al. (2008). This might indicate that prosocial behavior and empathy share a common origin, but that they are also driven by their unique biological and environmental processes.

We subsequently addressed the question whether there was covariance between structure of the social brain, prosocial behavior, and empathy. We were primarily interested in brain regions that showed a consistent genetic factor, similar to what was observed for prosocial behavior and empathy. This was especially the case for the precuneus, for which we found strong influences of genetics on both surface area and cortical thickness. Indeed, cortical thickness of the precuneus was negatively associated with empathy. Findings from our bivariate analyses showed that decreased cortical thickness of the precuneus and increased empathy were, to a small extent, driven by overlapping genetic and unique environmental factors. Although the shared genetic variance was small, the formal test of this shared genetic relation provides evidence that genetic variance is correlated among both constructs, suggesting that some overlapping genetic factors drive variance in both cortical thickness of the precuneus and empathy.

Interestingly, previous functional neuroimaging studies have indicated a positive link between the precuneus and prosocial behavior and empathy (Masten et al., 2010; Rameson et al., 2011; van der Meulen et al., 2018), although structural neuroimaging findings showed mixed findings for boys and girls (Thijssen et al., 2015). Cortical thickness generally decreases across development, indicating advances in brain maturation (Mills et al., 2014; Wierenga et al., 2014). Although a lower value of cortical thickness does not necessarily reflect brain maturation, it might be interesting to investigate whether the negative association found in the current study indicates a link between a matured precuneus and increased prosocial behavior in middle childhood. Alternatively, our findings might hint at the possibility that more empathic individuals have an overall lower cortical thickness (also proposed by Ferschmann et al. (2019)). This question should be tested in future research using longitudinal twin analyses.

Prior studies have pinpointed the precuneus as an important region for evaluating both the self and other persons (Ochsner et al., 2005; Pfeifer et al., 2007). Possibly, the precuneus plays a crucial role in differentiating between self and other, thereby facilitating perspective taking in a social situation. In addition, the precuneus is involved in autobiographical memory (for review see Cavanna and Trimble (2006)), which might enable an accurate recall of one’s capability to help another in distress. The involvement of the precuneus in both perspective taking and recall of one’s own capabilities might make the precuneus an essential brain region for prosocial behavior in childhood. It should be noted that our findings regarding the precuneus were more similar to the patterns observed for cuneus and lingual gyrus (which were included as control regions), than to our findings for other regions in the social brain network. This emphasizes the need to better understand the role of the precuneus in the social brain network, and to determine whether biological and environmental influences on brain structure are similar for neighboring brain regions (i.e. precuneus and lingual gyrus), or for brain regions that are part of the same network (i.e. precuneus and mPFC). Furthermore, our finding in this specific age range is particularly important to better understand the starting point of the large-scale brain development of adolescence (Mills et al., 2016; Vijayakumar et al., 2016; Wierenga et al., 2014). Future research should examine brain-behavior associations in more detail as well as moderating factors that can influence these relations. Previous work indicates, for example, faster neural maturation of both surface area and cortical thickness of social brain regions in girls (Mills et al., 2014; Mutlu et al., 2013), and others reported sex differences in prosocial behavior (Knafo-Noam et al., 2015). It is therefore possible that boys and girls in middle childhood show different or opposing directions in brain-behavior associations. Since we controlled for sex in the current study, possible sex effects in brain-behavior associations (as found by Thijssen et al. (2015)) may have been obscured, resulting in mostly non-significant associations between structure of the social brain and prosocial behavior and empathy.

### 4.3 Limitations

The current study had several limitations that should be addressed in future research. First, although we differentiated between prosocial behavior and empathy we did not further account for other constructs related to prosocial behavior (such as perspective taking and mentalizing). Although these constructs commonly associated with the social brain and prosocial behavior, we did not include behavioral measures of perspective taking in our design. Within prosocial behavior, researchers distinguish between context-specific costly prosocial behavior (helping or sharing at the cost of one-self) and non-costly prosocial behavior (helping and sharing to benefit others but at no cost for self; Fehr et al. (2008)), and general prosocial behavior (the intention to help, comfort, or share with others). For the current study, we have chosen to investigate prosocial behavior across contexts, rather than a specific situation, thereby providing a more general perspective on prosocial behavior but more limited in terms of potential response biases of the informants. Future research should aim to disentangle genetic and environmental effects for various types of prosocial behavior, in order to achieve a more comprehensive understanding of this multidimensional construct.

Second, we limited our selection of regions of interest in the social brain to four key regions (mPFC, TPJ, pSTS, and precuneus). Although this ROI driven approach increases statistical power, for a more comprehensive understanding of genetic and environmental influences on the social brain it might be interesting to also include regions such as the anterior cingulate cortex (ACC), amygdala, and anterior insula in future studies, as these regions are also involved in social cognition and behavior (Blakemore, 2008). Third, the current study is cross-sectional and therefore no interpretations can be made regarding the relationship between structure of the social brain and prosocial behavior across development. In order to better understand influences of genetics and environment on brain development and brain-behavior associations over time a longitudinal design is required (Brans et al., 2010; Mills et al., 2014; Shaw et al., 2006). By employing multi-modality approaches (i.e. combining structural and functional MRI with behavioral observations and parent report) we might also increase our understanding of the mediating mechanisms through which genes might influence the brain, which in turn can influence behavior. Finally, although our results indicate influence of familial factors on some structural measures of the social brain, we could not always differentiate between genetic and/or shared environmental factors. In addition, we did not investigate underlying neurobiological mechanisms that influence susceptibility to the environment. Although the TPJ, which was most sensitive to environmental influences, correlates in functional brain imaging studies with social behavior, such as in tasks tapping into reflected self-concept (Van der Cruijsen et al., 2019) or peer influence (Van Hoorn et al., 2016b), we currently do not know which neurobiological mechanisms drive increased environmental susceptibility. Our initial findings should therefore be taken as a starting point for future research on larger samples using longitudinal measurements.

### 4.4 Conclusion

The current study contributes to the current theoretical framework by investigating the influence of genetics and environment on brain regions that are of particular interest for (pro)social behavior. Moreover, brain-behavior relationships were studied in a relatively young sample, around or prior to gray matter changes in adolescence. This twin-study confirmed the hypothesis that regions of the social brain showed influences of shared environmental factors. Our initial findings show that there might be relatively more influence of shared environment on cortical thickness than on surface area, but these findings should be assessed in more detail in future research on larger samples using longitudinal measurements. In addition, we found that especially the TPJ might be more susceptible to environmental and social influences. Structural properties of the precuneus showed strong influence of genetics, which partly overlapped with genetic influence on parent-reported empathy, indicating that similar biological and environmental processes drive variance in this brain-behavior relationship. An important question for future research is whether behavioral interventions aimed at increasing prosocial behavior have an impact on the developmental trajectory of the social brain regions, which would provide stronger evidence for an impact of environment on brain and behavioral development.

## Declaration of Competing Interest

The authors declare no competing financial interest.
