# Supplementary material for: Genetic and environmental influences on structure of the social brain in childhood
Source: Dev Cogn Neurosci. 2020 May 5;44:100782. doi: 10.1016/j.dcn.2020.100782 (PMC7374548; doi:10.1016/j.dcn.2020.100782)
Supplement: Supplementary file 1 [file mmc1.docx]

**Supplementary Materials**

**Genetic and environmental influences on structure of the social brain in childhood**

**Mara van der Meulen**^1,2,3^, **Lara M. Wierenga**^1,2,3^, **Michelle Achterberg**^1,2,3^, **Nadieh** **Drenth**^1,4^, **Marinus H. van** **IJzendoorn**^1,5,6^, **Eveline A.** **Crone**^1,2,3^

^1^ Leiden Consortium on Individual Development, Leiden University, The Netherlands
^2^ Institute of Psychology, Leiden University, The Netherlands
^3^ Leiden Institute for Brain and Cognition, Leiden University, The Netherlands
^4^ Department of Radiology, Leiden University Medical Center, The Netherlands
^5^ Department of Psychology, Education and Child Studies, Erasmus University, The Netherlands
^6^ School of Clinical Medicine, University of Cambridge, UK

**Corresponding author**: Mara van der Meulen, Faculty of Social and Behavioural Sciences, Leiden University, Wassenaarseweg 52, 2333 AK Leiden, The Netherlands.
Tel: +31 71 527 3510, E-mail: [m.van.der.meulen@fsw.leidenuniv.nl](mailto:m.van.der.meulen@fsw.leidenuniv.nl)

**Figure S1.** Visualisation of within-twin correlations for surface area (left) and cortical thickness (right) of mPFC, TPJ, pSTS, precuneus, cuneus and lingual gyrus.


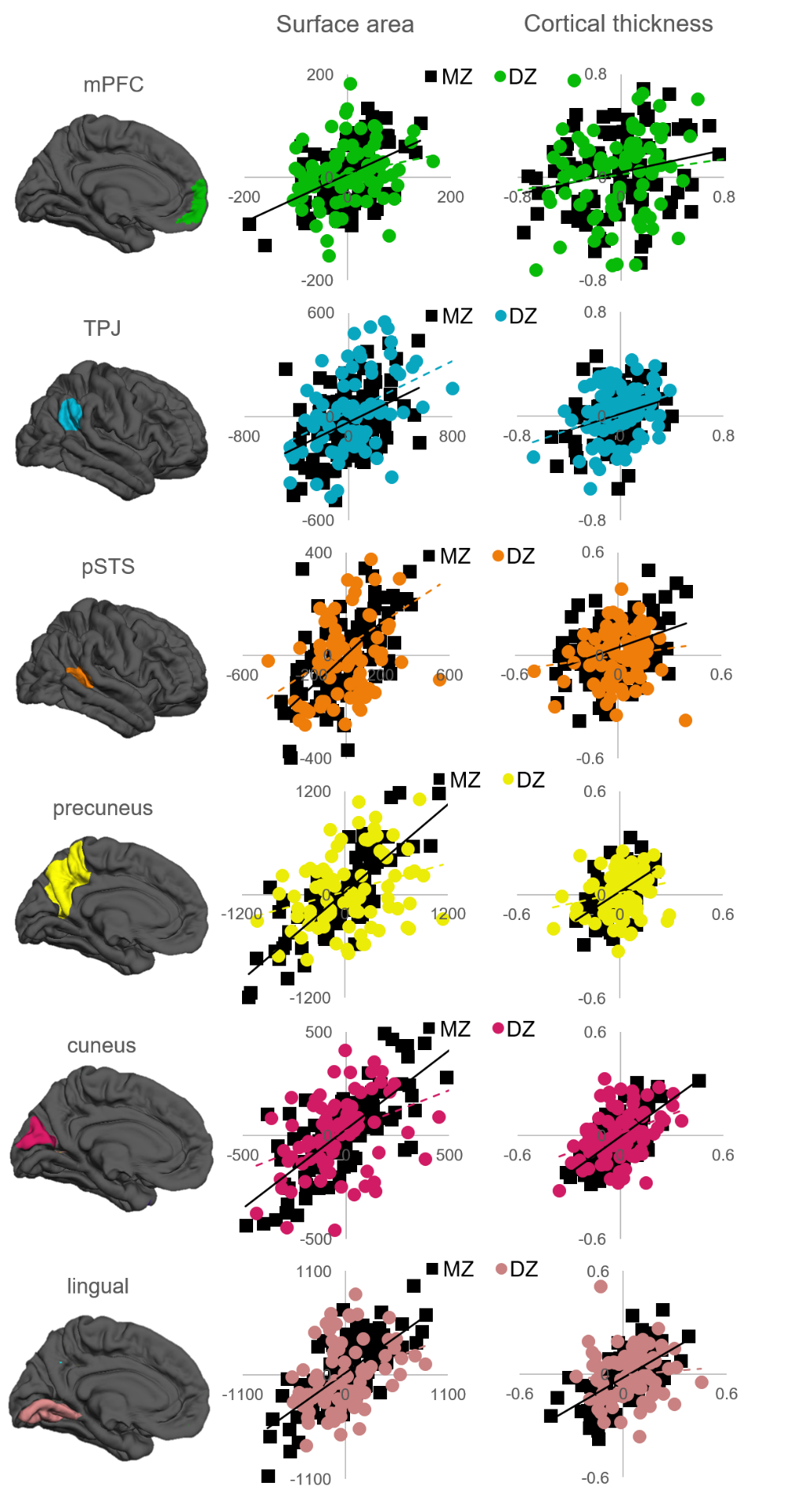


MZ = monozygotic; DZ = dizygotic

**Table S1.** Questionnaire structure after principal component analysis.

|  | Original questionnaire |
| --- | --- |
| *Prosocial subscale* |  |
| Considerate of other people's feelings | SDQ |
| Shares readily with other children | SDQ |
| Helpful if someone is hurt | SDQ |
| Kind to younger children | SDQ |
| Often volunteers to help others | SDQ |
| Will try to comfort or reassure another in distress | MC |
| Likely to offer toys or candy to a crying playmate even without parental suggestion | MC |
| Likely to show spontaneous nurturing and care-giving behavior toward an animal | MC |
| Can tell at just a glance how others are feeling | MC |
| Likely to ask, "What's wrong?" when seeing someone in distress | MC |
| Will feel sorry for other people who are hurt, sick, or unhappy | MC |
|  |  |
| *Empathy subscale* |  |
| Acts upset when he/she sees a hurt animal | MC |
| Rarely cries or looks upset when watching a sad TV show | MC |
| Gets angry at aggressor, "Bad Guy", who hurts a TV character | MC |
| Is upset by stories in which characters are hurt or die | MC |
| Is not likely to become upset if a playmate cries | MC |
|  |  |
| May occassionally tease a pet if unsupervised | MC |
| Feels good when good things happen to movie characters | MC |

Note. SDQ = Strenghts and Difficulties Questionnaire; MC = My Child Questionnaire

**Table S2.** Correlations between mean factor scores (prosocial and empathy subscale) and original questionnaires (SDQ and MC). Correlation coefficients under diagonal are for sample A, coefficients above diagonal are for sample B.

|  |  | *Mean factor scores* | | *Original questionnaires* | |
| --- | --- | --- | --- | --- | --- |
|  |  | Prosocial behavior | Empathy | SDQ | MC |
| *Mean factor scores* | Prosocial behavior | - | .36*** | .93*** | .75*** |
|  | Empathy | .33*** | - | .28*** | .84*** |
| *Original questionnaires* | SDQ | .92*** | .22** | - | .59*** |
|  | MC | .69*** | .86*** |  | - |

SDQ = Strenghts and Difficulties Questionnaire; MC = My Child Questionnaire

* *p* < .05; ** *p* < .01; *** *p* < .001.

Correlation coefficients did not differ significantly between sample A and sample B.

**Table S3.** Results of regression analyses with age, IQ and sex as predictors.

|  | Sample A | | |  | Sample B | | |
| --- | --- | --- | --- | --- | --- | --- | --- |
| *Surface Area* | B | SE | *β* |  | B | SE | *β* |
| mPFC |  |  |  |  |  |  |  |
| (Constant) | 485.72 | 56.46 |  |  | 431.53 | 57.46 |  |
| Age | 11.48 | 5.8 | **.12*** |  | 8.67 | 5.87 | 0.09 |
| IQ | 0.39 | 0.33 | 0.07 |  | 1.08 | 0.34 | **.21**** |
| Sex (dummy var) | -53.27 | 7.77 | **-.42***** |  | -40.81 | 8.14 | **-.33***** |
|  |  |  |  |  |  |  |  |
| TPJ |  |  |  |  |  |  |  |
| (Constant) | 1333.4 | 218.5 |  |  | 1224.6 | 240.4 |  |
| Age | 17.37 | 22.43 | 0.05 |  | 6.58 | 24.56 | 0.02 |
| IQ | 1.4 | 1.28 | 0.7 |  | 2.98 | 1.4 | **.14*** |
| Sex (dummy var) | -234.1 | 30.07 | **-.47***** |  | -195.3 | 34.05 | **-.37***** |
|  |  |  |  |  |  |  |  |
| pSTS |  |  |  |  |  |  |  |
| (Constant) | 1220.1 | 186.3 |  |  | 1414.6 | 189.4 |  |
| Age | 17.61 | 19.13 | 0.06 |  | -4.27 | 19.36 | -0.01 |
| IQ | 1.2 | 1.09 | 0.07 |  | 0.81 | 1.1 | 0.05 |
| Sex (dummy var) | -180.5 | 25.64 | **-.43***** |  | -162.6 | 26.83 | **-.39***** |
|  |  |  |  |  |  |  |  |
| precuneus |  |  |  |  |  |  |  |
| (Constant) | 3443.1 | 457.5 |  |  | 2780.2 | 445.9 |  |
| Age | 32.59 | 46.97 | 0.04 |  | 51.06 | 45.56 | 0.07 |
| IQ | 4.88 | 2.68 | 0.11 |  | 9.47 | 2.59 | **.23***** |
| Sex (dummy var) | -437.4 | 62.97 | **-.43***** |  | -404.1 | 63.15 | **-.40***** |
|  |  |  |  |  |  |  |  |
| Cuneus |  |  |  |  |  |  |  |
| (Constant) | 1365.7 | 207.3 |  |  | 1333.8 | 193.9 |  |
| Age | 0.12 | 21.28 | 0 |  | 8.35 | 19.81 | 0.03 |
| IQ | 2.63 | 1.21 | 0.13 |  | 2.39 | 1.13 | **0.14*** |
| Sex (dummy var) | -126.7 | 28.53 | **-.29***** |  | -147.4 | 27.46 | **-0.35***** |
|  |  |  |  |  |  |  |  |
| Lingual |  |  |  |  |  |  |  |
| (Constant) | 2324.8 | 364.5 |  |  | 2089.9 | 329.8 |  |
| Age | 51.08 | 37.42 | 0.09 |  | 40.42 | 33.7 | 0.08 |
| IQ | 3.9 | 2.13 | 0.12 |  | 7.24 | 1.92 | **0.25***** |
| Sex (dummy var) | -243.4 | 50.17 | **-0.31***** |  | -214.7 | 46.72 | **-0.3***** |
|  |  |  |  |  |  |  |  |
| *Cortical Thickness* | |  |  |  |  |  |  |
| mPFC |  |  |  |  |  |  |  |
| (Constant) | 4.1 | 0.31 |  |  | 4.05 | 0.32 |  |
| Age | -0.06 | 0.03 | -0.13 |  | -0.04 | 0.04 | -0.08 |
| IQ | -0.001 | 0.002 | -0.025 |  | -0.002 | 0.002 | -0.07 |
| Sex (dummy var) | 0.02 | 0.04 | 0.03 |  | -0.009 | 0.05 | -0.01 |

**Table S3.** Continued.

|  | Sample A | | |  | Sample B | | |
| --- | --- | --- | --- | --- | --- | --- | --- |
| TPJ |  |  |  |  |  |  |  |
| (Constant) | 3.14 | 0.19 |  |  | 2.989 | 0.197 |  |
| Age | -0.02 | 0.02 | -0.08 |  | -0.01 | 0.02 | -0.03 |
| IQ | 0 | 0.001 | -0.02 |  | 0 | 0.001 | 0.03 |
| Sex (dummy var) | 0.08 | 0.03 | **.197***** |  | 0.02 | 0.03 | 0.04 |
|  |  |  |  |  |  |  |  |
| pSTS |  |  |  |  |  |  |  |
| (Constant) | 3.18 | 0.17 |  |  | 3.35 | 0.16 |  |
| Age | -0.02 | 0.02 | -0.08 |  | -0.03 | 0.02 | -0.14 |
| IQ | 0 | 0.001 | 0.01 |  | 0 | 0.001 | -0.032 |
| Sex (dummy var) | 0.03 | 0.02 | 0.09 |  | -0.001 | 0.02 | -0.002 |
|  |  |  |  |  |  |  |  |
| precuneus |  |  |  |  |  |  |  |
| (Constant) | 3.11 | 0.13 |  |  | 3.36 | 0.12 |  |
| Age | -0.03 | 0.01 | **-.14*** |  | -0.04 | 0.01 | **-.24***** |
| IQ | 0 | 0.001 | 0.01 |  | -0.001 | 0.001 | -0.09 |
| Sex (dummy var) | 0.03 | 0.02 | 0.1 |  | 0.01 | 0.02 | 0.03 |
|  |  |  |  |  |  |  |  |
| Cuneus |  |  |  |  |  |  |  |
| (Constant) | 2.62 | 0.14 |  |  | 2.54 | 0.14 |  |
| Age | -0.04 | 0.01 | **-.20**** |  | -0.02 | 0.02 | -0.12 |
| IQ | 0 | 0.001 | -0.02 |  | -0.001 | 0.001 | -0.08 |
| Sex (dummy var) | -0.01 | 0.02 | -.04 |  | -0.02 | 0.02 | -.06 |
|  |  |  |  |  |  |  |  |
| Lingual |  |  |  |  |  |  |  |
| (Constant) | 2.77 | 0.13 |  |  | 2.52 | 0.15 |  |
| Age | -0.06 | 0.01 | **-.28***** |  | -0.03 | 0.02 | -0.12 |
| IQ | 0 | 0.001 | -0.01 |  | 0 | 0.001 | -.02 |
| Sex (dummy var) | -0.01 | 0.02 | -.03 |  | -0.03 | 0.02 | -.08 |
|  |  |  |  |  |  |  |  |
| *Behavior* |  |  |  |  |  |  |  |
| Prosocial behavior | |  |  |  |  |  |  |
| (Constant) | 4.96 | 0.44 |  |  | 4.63 | 0.47 |  |
| Age | -0.05 | 0.04 | -0.07 |  | 0.03 | 0.05 | 0.04 |
| IQ | -0.003 | 0.003 | -0.08 |  | -0.01 | 0.003 | **-.15*** |
| Sex (dummy var) | 0.19 | 0.06 | **.06**** |  | 0.23 | 0.07 | **.22**** |
|  |  |  |  |  |  |  |  |
| Empathy |  |  |  |  |  |  |  |
| (Constant) | 2.3 | 0.68 |  |  | 2.08 | 0.65 |  |
| Age | 0.07 | 0.07 | 0.06 |  | 0.13 | 0.07 | 0.12 |
| IQ | -0.001 | 0.004 | -0.01 |  | -0.003 | 0.004 | -0.05 |
| Sex (dummy var) | 0.43 | 0.09 | **.30***** |  | 0.45 | 0.09 | **.30***** |

* *p* < .05; ** *p* < .01; *** *p* < .001. Significant associations are indicated by bold font.

**Table S4.** Comparison of univariate ACE models for bilateral structural properties of regions of the social brain, prosocial behavior, and empathy.

| **Outcome variable** | **model** | **A²** | **C²** | **E²** | **LRT** | **AIC** |
| --- | --- | --- | --- | --- | --- | --- |
| *Surface area* |  |  |  |  |  |  |
| mPFC | ACE | 0.48 | 0.00 | 0.52 |  | 3187.77 |
|  | **AE*** | **0.48** | **-** | **0.52** | **0.00** | **3185.77** |
|  | CE | - | 0.34 | 0.66 | 6.10 | 3191.87 |
|  | E | - | - | 1.00 | > 22.06 | 3211.92 |
|  |  |  |  |  |  |  |
| TPJ | ACE | 0.25 | 0.21 | 0.53 |  | 4185.52 |
|  | **AE*** | **0.50** | **-** | **0.50** | **1.23** | **4184.75** |
|  | **CE*** | **-** | **0.39** | **0.61** | **1.16** | **4184.68** |
|  | E | - | - | 1.00 | > 30.43 | 4213.178 |
|  |  |  |  |  |  |  |
|  | ACE | 0.61 | 0.04 | 0.35 |  | 4036.18 |
| pSTS | **AE*** | **0.65** | **-** | **0.35** | **0.05** | **4034.23** |
|  | CE | - | 0.49 | 0.51 | 9.76 | 4043.94 |
|  | E | - | - | 1.00 | > 50.30 | 4092.24 |
|  |  |  |  |  |  |  |
|  | ACE | 0.81 | 0.00 | 0.19 |  | 1286.61 |
| Precuneus | **AE*** | **0.81** | **-** | **0.19** | **0.00** | **1284.61** |
|  | CE | - | 0.59 | 0.41 | 37.27 | 1321.88 |
|  | E | - | - | 1.00 | > 76.32 | 1396.20 |
|  |  |  |  |  |  |  |
|  | ACE | 0.70 | 0.02 | 0.29 |  | 716.43 |
| Cuneus | **AE*** | **0.71** | **-** | **0.29** | **0.01** | **714.44** |
|  | CE | - | 0.57 | 0.43 | 15.02 | 729.45 |
|  | E | - | - | 1.00 | > 71.03 | 798.49 |
|  |  |  |  |  |  |  |
|  | ACE | 0.70 | 0.01 | 0.29 |  | 4417.33 |
| Lingual | **AE*** | **0.71** | **-** | **0.29** | **-0.01** | **4415.32** |
|  | CE | - | 0.57 | 0.43 | 15.67 | 4430.99 |
|  | E | - | - | 1.00 | > 69.92 | 4498.91 |
|  |  |  |  |  |  |  |
| *Cortical thickness* | |  |  |  |  |  |
| mPFC | ACE | 0.17 | 0.05 | 0.78 |  | -540.53 |
|  | **AE*** | **0.23** | **-** | **0.77** | **0.05** | **-542.48** |
|  | **CE*** | **-** | **0.18** | **0.82** | **0.34** | **-542.19** |
|  | E | - | - | 1.00 | > 5.79 | -538.40 |
|  |  |  |  |  |  |  |
| TPJ | ACE | 0.09 | 0.23 | 0.68 |  | -900.45 |
|  | **AE*** | **0.36** | **-** | **0.64** | **1.16** | **-901.29** |
|  | **CE*** | **-** | **0.30** | **0.70** | **0.12** | **-902.33** |
|  | E | - | - | 1.00 | > 15.42 | -887.87 |

**Table S4.** Continued.

| **Outcome variable** | **model** | **A²** | **C²** | **E²** | **LRT** | **AIC** |
| --- | --- | --- | --- | --- | --- | --- |
| pSTS | ACE | 0.23 | 0.05 | 0.72 |  | -1005.85 |
|  | **AE*** | **0.29** | **-** | **0.71** | **0.04** | **-1007.81** |
|  | **CE*** | **-** | **0.25** | **0.75** | **0.59** | **-1007.26** |
|  | E | - | - | 1.00 | > 11.37 | -997.89 |
|  |  |  |  |  |  |  |
| Precuneus | ACE | 0.55 | 0.00 | 0.45 |  | -1267.59 |
|  | **AE*** | **0.55** | **-** | **0.45** | **0.00** | **-1269.59** |
|  | CE | - | 0.36 | 0.64 | 9.75 | -1259.84 |
|  | E | - | - | 1.00 | > 25.11 | -1236.73 |
|  |  |  |  |  |  |  |
| Cuneus | ACE | 0.73 | 0.00 | 0.27 |  | -1197.10 |
|  | **AE*** | **0.73** | **-** | **0.27** | **< .001** | **-1199.10** |
|  | CE | - | 0.53 | 0.47 | 20.14 | -1178.96 |
|  | E | - | - | 1.00 | > 60.18 | -1120.78 |
|  |  |  |  |  |  |  |
| Lingual | ACE | 0.56 | 0.00 | 0.44 |  | -1129.61 |
|  | **AE*** | **0.56** | **-** | **0.44** | **0.00** | **-1131.61** |
|  | CE | - | 0.37 | 0.63 | 14.15 | -1117.47 |
|  | E | - | - | 1.00 | > 25.75 | -1093.72 |
|  |  |  |  |  |  |  |
| *Parent report* | |  |  |  |  |  |
| Prosocial behavior | ACE | 0.45 | 0 | 0.55 |  | -320.56 |
|  | **AE*** | **0.45** | **-** | **0.55** | **< .001** | **-322.56** |
|  | CE | - | 0.28 | 0.72 | 10.94 | -311.62 |
|  | E | - | - | 1 | > 20.06 | -293.57 |
|  |  |  |  |  |  |  |
| Empathy | ACE | 0.59 | 0.15 | 0.27 |  | -61.17 |
|  | **AE*** | **0.74** | **-** | **0.26** | **0.8** | **-62.38** |
|  | CE | - | 0.61 | 0.39 | 17.44 | -45.74 |
|  | E | - | - | 1 | > 114.86 | 67.12 |

Best fitting models are indicated by an asterisk (*) and bold font.

**Table S5.** Comparison of univariate ACE models for structural properties of left-hemisphere regions of the social brain.

| **Outcome variable** | **rMZ** | **rDZ** | **model** | **A²** | **C²** | **E²** | **LRT** | **AIC** |
| --- | --- | --- | --- | --- | --- | --- | --- | --- |
| *Surface area* |  |  |  |  |  |  |  |  |
| mPFC | .30** | .28* | *95% CI* | *(†-0.46)* | *(0.00-0.41)* | *(0.53-0.84)* |  |  |
|  |  |  | ACE | 0.08 | 0.22 | 0.70 |  | 3129.94 |
|  |  |  | **AE*** | **0.34** | **-** | **0.66** | **1.03** | **3128.96** |
|  |  |  | **CE*** | **-** | **0.28** | **0.72** | **0.09** | **3128.03** |
|  |  |  | E | - | - | 1.00 | > 14.29 | 3141.26 |
|  |  |  |  |  |  |  |  |  |
| TPJ | .48*** | 0.20 | *95% CI* | *(0.12-0.60)* | *(†-0.28)* | *(0.39-0.68)* |  |  |
|  |  |  | ACE | 0.45 | 0.04 | 0.51 |  | 4335.69 |
|  |  |  | **AE*** | **0.49** | **-** | **0.51** | **-0.32** | **4333.37** |
|  |  |  | CE | - | 0.34 | 0.66 | 6.18 | 4339.88 |
|  |  |  | E | - | - | 1.00 | > 22.44 | 4360.32 |
|  |  |  |  |  |  |  |  |  |
| pSTS | .58*** | .24* | *95% CI* | *(0.46-0.70)* | *(†-0.25)* | *(0.30-0.54)* |  |  |
|  |  |  | ACE | 0.59 | 0.00 | 0.41 |  | 4223.10 |
|  |  |  | **AE*** | **0.59** | **-** | **0.41** | **< .001** | **4221.10** |
|  |  |  | CE | - | 0.42 | 0.58 | 10.96 | 4232.06 |
|  |  |  | E | - | - | 1.00 | > 35.56 | 4264.61 |
|  |  |  |  |  |  |  |  |  |
| Precuneus | .72*** | .23* | *95% CI* | *(0.59-0.77)* | *(†-0.23)* | *(0.23-0.42)* |  |  |
|  |  |  | ACE | 0.69 | 0.00 | 0.31 |  | 1350.77 |
|  |  |  | **AE*** | **0.69** | **-** | **0.31** | **0.00** | **1348.77** |
|  |  |  | CE | - | 0.52 | 0.48 | 19.09 | 1367.86 |
|  |  |  | E | - | - | 1.00 | > 56.89 | 1422.45 |
|  |  |  |  |  |  |  |  |  |
| Cuneus | .62*** | .31** | *95% CI* | *(0.00-0.60)* | *(0.00-0.52)* | *(0.33-0.55)* |  |  |
|  |  |  | ACE | 0.43 | 0.14 | 0.43 |  | 4108.11 |
|  |  |  | AE* | 0.58 | - | 0.42 | **0.35** | **4106.45** |
|  |  |  | CE | - | 0.50 | 0.50 | 3.85 | 4109.96 |
|  |  |  | E | - | - | 1.00 | > 51.46 | 4159.42 |
|  |  |  |  |  |  |  |  |  |
| Lingual | .71*** | 0.21 | *95% CI* | *(0.53-0.78)* | *(†-0.23)* | *(0.24-0.43)* |  |  |
|  |  |  | ACE | 0.66 | 0.02 | 0.32 |  | 4475.71 |
|  |  |  | **AE*** | **0.68** | **-** | **0.32** | **-0.29** | **4473.43** |
|  |  |  | CE | - | 0.51 | 0.49 | 18.07 | 4491.78 |
|  |  |  | E | - | - | 1.00 | > 53.42 | 4543.20 |

**Table S5.** Continued.

| **Outcome variable** | **rMZ** | **rDZ** | **model** | **A²** | **C²** | **E²** | **LRT** | **AIC** |
| --- | --- | --- | --- | --- | --- | --- | --- | --- |
| *Cortical thickness* | | |  |  |  |  |  |  |
| mPFC | .21* | .21 | *95% CI* | *(0.00-0.39)* | *(0.00-0.33)* | *(0.61-0.95)* |  |  |
|  |  |  | ACE | 0.01 | 0.19 | 0.80 |  | -358.41 |
|  |  |  | **AE*** | **0.24** | **-** | **0.76** | **0.72** | **-359.69** |
|  |  |  | **CE*** | **-** | **0.20** | **0.80** | **< .001** | **-360.41** |
|  |  |  | E | - | - | 1.00 | > 6.40 | -355.29 |
|  |  |  |  |  |  |  |  |  |
| TPJ | .26** | 0.09 | *95% CI* | *(0.00-0.40)* | *(0.00-0.29)* | *(0.60-0.94)* |  |  |
|  |  |  | ACE | 0.24 | 0.00 | 0.76 |  | -762.17 |
|  |  |  | **AE*** | **0.24** | **-** | **0.76** | **0.00** | **-764.17** |
|  |  |  | **CE*** | **-** | **0.18** | **0.82** | **1.19** | **-762.98** |
|  |  |  | E | - | - | 1.00 | > 5.91 | -759.07 |
|  |  |  |  |  |  |  |  |  |
| pSTS | .32** | .02 | *95% CI* | *(0.00-0.40)* | *(0.00-0.27)* | *(0.60-0.92)* |  |  |
|  |  |  | ACE | 0.24 | 0.00 | 0.76 |  | -884.01 |
|  |  |  | **AE*** | **0.24** | **-** | **0.76** | **0.00** | **-886.01** |
|  |  |  | **CE*** | **-** | **0.17** | **0.83** | **1.98** | **-884.03** |
|  |  |  | E | - | - | 1.00 | > 5.59 | -880.44 |
|  |  |  |  |  |  |  |  |  |
| Precuneus | .43*** | .25* | *95% CI* | *(0.00-0.57)* | *(†-0.42)* | *(0.43-0.74)* |  |  |
|  |  |  | ACE | 0.40 | 0.04 | 0.56 |  | -1206.20 |
|  |  |  | **AE*** | **0.44** | **-** | **0.56** | **0.04** | **-1208.16** |
|  |  |  | CE | - | 0.34 | 0.66 | 2.55 | -1205.65 |
|  |  |  | E | - | - | 1.00 | > 22.16 | -1185.49 |
|  |  |  |  |  |  |  |  |  |
| Cuneus | .60*** | .23* | *95% CI* | *(0.31-0.70)* | *(0.00 - 0.22)* | *(0.30-0.54)* |  |  |
|  |  |  | ACE | 0.59 | 0.00 | 0.41 |  | -1044.11 |
|  |  |  | **AE*** | **0.59** | **-** | **0.41** | **0.00** | **-1046.11** |
|  |  |  | CE | - | 0.41 | 0.59 | 12.05 | -1034.06 |
|  |  |  | E | - | - | 1.00 | > 33.68 | -1002.38 |
|  |  |  |  |  |  |  |  |  |
| Lingual | .60*** | .09 | *95% CI* | *(0.35-0.68)* | *(0.00-0.16)* | *(0.32-0.59)* |  |  |
|  |  |  | ACE | 0.56 | 0.00 | 0.44 |  | -1131.25 |
|  |  |  | **AE*** | **0.56** | **-** | **0.44** | **0.00** | **-1133.25** |
|  |  |  | CE | - | 0.37 | 0.63 | 13.97 | -1119.28 |
|  |  |  | E | - | - | 1.00 | > 26.14 | -1095.14 |

Best fitting models are indicated by an asterisk (*) and bold font.

**Table S6.** Comparison of univariate ACE models for structural properties of right-hemisphere regions of the social brain.

| **Outcome variable** | **rMZ** | **rDZ** | **model** | **A²** | **C²** | **E²** | **LRT** | **AIC** |
| --- | --- | --- | --- | --- | --- | --- | --- | --- |
| *Surface area* |  |  |  |  |  |  |  |  |
| mPFC | .30** | .11 | *95% CI* | *(0.09-0.44)* | *(†-0.30)* | *(0.57-0.91)* |  |  |
|  |  |  | ACE | 0.27 | 0.00 | 0.73 |  | 3454.51 |
|  |  |  | **AE*** | **0.27** | **-** | **0.73** | **0.00** | **3452.51** |
|  |  |  | **CE*** | **-** | **0.19** | **0.81** | **1.76** | **3454.27** |
|  |  |  | E | - | - | 1.00 | > 6.93 | 3459.20 |
|  |  |  |  |  |  |  |  |  |
| TPJ | .23* | .34** | *95% CI* | *(†-0.42)* | *(0.00-0.42)* | *(0.56-0.84)* |  |  |
|  |  |  | ACE | 0.06 | 0.23 | 0.70 |  | 4351.62 |
|  |  |  | **AE*** | **0.33** | **-** | **0.67** | **2.45** | **4352.07** |
|  |  |  | **CE*** | **-** | **0.29** | **0.71** | **-0.35** | **4349.27** |
|  |  |  | E | - | - | 1.00 | > 13.13 | 4363.20 |
|  |  |  |  |  |  |  |  |  |
| pSTS | .40*** | .33** | *95% CI* | *(0.00-0.55)* | *(0.00-0.46)* | *(0.46-0.74)* |  |  |
|  |  |  | ACE | 0.25 | 0.16 | 0.59 |  | 4106.92 |
|  |  |  | **AE*** | **0.43** | **-** | **0.57** | **0.62** | **4105.53** |
|  |  |  | **CE*** | **-** | **0.35** | **0.65** | **1.02** | **4105.94** |
|  |  |  | E | - | - | 1.00 | > 23.74 | 4127.68 |
|  |  |  |  |  |  |  |  |  |
| Precuneus | .75*** | .29** | *95% CI* | *(0.65-0.81)* | *(†-0.23)* | *(0.19-0.35)* |  |  |
|  |  |  | ACE | 0.74 | 0.00 | 0.26 |  | 1357.84 |
|  |  |  | **AE*** | **0.74** | **-** | **0.26** | **0.00** | **1355.84** |
|  |  |  | CE | - | 0.55 | 0.45 | 23.97 | 1379.81 |
|  |  |  | E | - | - | 1.00 | > 65.23 | 1443.04 |
|  |  |  |  |  |  |  |  |  |
| Cuneus | .66*** | .35** | *95% CI* | *(0.49-0.73)* | *(†-0.38)* | *(0.26-0.47)* |  |  |
|  |  |  | ACE | 0.62 | 0.03 | 0.35 |  | 4114.86 |
|  |  |  | **AE*** | **0.65** | **-** | **0.35** | **0.02** | **4112.88** |
|  |  |  | CE | - | 0.51 | 0.49 | 10.06 | 4122.92 |
|  |  |  | E | - | - | 1.00 | > 54.32 | 4175.23 |
|  |  |  |  |  |  |  |  |  |
| Lingual | .62*** | .40*** | *95% CI* | *(0.00-0.58)* | *(0.00-0.56)* | *(0.30-0.52)* |  |  |
|  |  |  | ACE | 0.35 | 0.25 | 0.40 |  | 4510.12 |
|  |  |  | **AE*** | **0.61** | **-** | **0.39** | **1.21** | **4509.33** |
|  |  |  | **CE*** | **-** | **0.53** | **0.47** | **3.27** | **4511.39** |
|  |  |  | E | - | - | 1.00 | > 58.11 | 4567.50 |

**Table S6.** Continued.

| **Outcome variable** | **rMZ** | **rDZ** | **model** | **A²** | **C²** | **E²** | **LRT** | **AIC** |
| --- | --- | --- | --- | --- | --- | --- | --- | --- |
| *Cortical Thickness* | |  |  |  |  |  |  |  |
| mPFC | 0.17 | .02 | *95% CI* | *(0.00-0.31)* | *(†-0.22)* | *(0.68-1.00)* |  |  |
|  |  |  | ACE | 0.14 | 0.00 | 0.86 |  | -419.77 |
|  |  |  | AE | 0.14 | - | 0.86 | 0.00 | -421.772 |
|  |  |  | CE | - | 0.09 | 0.91 | 0.79 | -420.987 |
|  |  |  | **E*** | **-** | **-** | **1.00** | **< 2.31** | **-421.462** |
|  |  |  |  |  |  |  |  |  |
| TPJ | 0.15 | .40*** | *95% CI* | *(0.00-0.28)* | *(0.03-0.40)* | *(0.60-0.87)* |  |  |
|  |  |  | ACE | 0.00 | 0.27 | 0.73 |  | -808.58 |
|  |  |  | **AE** | 0.28 | **-** | 0.72 | 4.48 | -806.10 |
|  |  |  | **CE*** | **-** | **0.27** | **0.73** | **< .001** | **-810.58** |
|  |  |  | E | - | - | 1.00 | > 9.21 | -798.89 |
|  |  |  |  |  |  |  |  |  |
| pSTS | .35*** | .24* | *95% CI* | *(0.00-0.43)* | *(0.00-0.42)* | *(0.56-0.85)* |  |  |
|  |  |  | ACE | 0.02 | 0.27 | 0.71 |  | -830.02 |
|  |  |  | **AE*** | **0.32** | **-** | **0.68** | **1.17** | **-830.85** |
|  |  |  | **CE*** | **-** | **0.29** | **0.71** | **0.00** | **-832.02** |
|  |  |  | E | - | - | 1.00 | > 14.59 | -818.26 |
|  |  |  |  |  |  |  |  |  |
| Precuneus | .46*** | .15 | *95% CI* | *(0.14-0.59)* | *(0.00-0.23)* | *(0.41-0.72)* |  |  |
|  |  |  | ACE | 0.45 | 0.00 | 0.55 |  | -1158.83 |
|  |  |  | **AE*** | **0.45** | **-** | **0.55** | **0.00** | **-1160.83** |
|  |  |  | CE | - | 0.29 | 0.71 | 6.43 | -1154.40 |
|  |  |  | E | - | - | 1 | > 15.98 | -1140.43 |
|  |  |  |  |  |  |  |  |  |
| Cuneus | .57*** | .28* | *95% CI* | *(0.22-0.69)* | *(0.00-0.30)* | *(0.31-0.54)* |  |  |
|  |  |  | ACE | 0.58 | 0.00 | 0.42 |  | -1077.56 |
|  |  |  | **AE*** | **0.58** | **-** | **0.42** | **0.00** | **-1079.56** |
|  |  |  | CE | - | 0.43 | 0.57 | 8.80 | -1070.75 |
|  |  |  | E | - | - | 1.00 | > 35.90 | -1036.85 |
|  |  |  |  |  |  |  |  |  |
| Lingual | .55*** | .17 | *95% CI* | *(0.28-0.66)* | *(0.00-0.20)* | *(0.34-0.61)* |  |  |
|  |  |  | ACE | 0.54 | 0.00 | 0.46 |  | -1122.17 |
|  |  |  | **AE*** | **0.54** | **-** | **0.46** | **0.00** | **-1124.17** |
|  |  |  | CE | - | 0.36 | 0.64 | 10.46 | -1113.71 |
|  |  |  | E | - | - | 1.00 | > 25.68 | -1090.03 |

**Table S7.** Phenotypic brain-behavior associations between structural brain measures (left and right hemisphere), prosocial behavior, and empathy.

|  | Left hemisphere | | | | Right hemisphere | | | |
| --- | --- | --- | --- | --- | --- | --- | --- | --- |
|  | Prosocial | | Empathy | | Prosocial | | Empathy | |
|  | *β* | *p* | *β* | *p* | *β* | *p* | *β* | *p* |
| *Surface Area* | |  |  |  |  |  |  |  |
| mPFC | .00 | .89 | .00 | .70 | .00 | .13 | .00 | .33 |
| TPJ | .00 | .78 | .00 | .26 | .00 | .85 | .00 | .08 |
| pSTS | .00 | .18 | .00 | .97 | .00 | .93 | .00 | .97 |
| Precuneus | .00 | .59 | .00 | .74 | .00 | .68 | .00 | .24 |
| Cuneus | .00 | .34 | .00 | .66 | .00 | .87 | .00 | .97 |
| Lingual | .00 | .27 | .00 | .71 | .00 | .92 | .00 | .46 |
|  |  |  |  |  |  |  |  |  |
| *Cortical Thickness* | |  |  |  |  |  |  |  |
| mPFC | .01 | .89 | .06 | .48 | .00 | .97 | -.06 | .52 |
| TPJ | -.14 | .20 | -.20 | .20 | **-.26** | **.02** | -.22 | .17 |
| pSTS | -.06 | .59 | -.03 | .89 | -.04 | .76 | -.02 | .93 |
| Precuneus | -.28 | .17 | **-.67** | **.01** | -.18 | .33 | **-.69** | **.00** |
| Cuneus | -.21 | .22 | -.13 | .53 | -.08 | .62 | **-.51** | **.02** |
| Lingual | .03 | .87 | -.32 | .15 | -.08 | .62 | **-.48** | **.04** |

Significant associations are indicated by bold font.

**Table S8.** Comparison of bivariate ACE models for bilateral cortical thickness of precuneus, prosocial behavior, and empathy.

| Model | -2LL | df | AIC | LRT | *p* |
| --- | --- | --- | --- | --- | --- |
| *Precuneus CT * Empathy* | |  |  |  |  |
| Saturated Cholesky | 48.19 | 656 | -1263.81 |  |  |
| ACE | 69.44 | 673 | -1276.56 | 21.25 | 0.22 |
| **AE*** | **69.80** | **676** | **-1282.20** | **0.36** | **0.95** |
| CE | 92.04 | 676 | -1259.96 | 25.05 | 0.00 |
| E | 191.84 | 679 | -1166.16 | 123.41 | 0.00 |
|  |  |  |  |  |  |
| *Prosocial behavior * Empathy* | | |  |  |  |
| Saturated Cholesky | 1473.00 | 944 | -415.00 |  |  |
| ACE | 1485.85 | 961 | -436.15 | 12.85 | 0.75 |
| **AE*** | **1488.26** | **964** | **-439.74** | **2.41** | **0.49** |
| CE | 1532.92 | 964 | -395.08 | 47.07 | 0.00 |
| E | 1663.80 | 967 | -270.20 | 177.95 | 0.00 |

Best fitting models are indicated by an asterisk (*) and bold font.

**Table S9.** Model statistics for best fitting bivariate model for bilateral cortical thickness of precuneus, prosocial behavior, and empathy.

|  | Squared standardized path loadings | | | | |  |  |
| --- | --- | --- | --- | --- | --- | --- | --- |
|  | path_11_ | path_12_ | | path_22_ | | *r_p_* | *r* |
| *Precuneus CT * Empathy* | | |  | |  |  |  |
| A | 0.56 (0.51-0.68) | 0.01 (0.00-0.06) | | 0.70 (0.60-0.78) | | 0.42 | -0.08 |
| C | - | - | | - | | - | - |
| E | 0.44 (0.32-0.51) | 0.01 (0.00-0.05) | | 0.28 (0.21-0.38) | | 0.58 | -0.20 |
|  |  | |  | |  |  |  |
| *Prosocial behavior * Empathy* | | | | |  |  |  |
| A | 0.45 (0.30-0.57) | 0.044 (0.01-0.13) | | 0.69 (0.61-0.75) | | 0.46 | 0.25 |
| C | - | - | | - | | - | - |
| E | 0.55 (0.43-0.70) | 0.05 (0.02-0.09) | | 0.22 (0.17-0.29) | | 0.54 | 0.42 |
